# Harnessing Big Data to Optimize an Algorithm for Rapid Diagnosis of Pulmonary Tuberculosis in a Real-World Setting

**DOI:** 10.3389/fcimb.2021.650163

**Published:** 2021-03-18

**Authors:** Jing Peng, Juan Song, Feng Wang, Peng Zuo, Yanjun Lu, Weiyong Liu, Lei Tian, Zhongju Chen, Yaowu Zhu, Xiong Wang, Na Shen, Xu Wang, Shiji Wu, Qin Yu, Bruce A. Vallance, Kevan Jacobson, Ziyong Sun, Hong Bing Yu

**Affiliations:** ^1^ Department of Laboratory Medicine, Tongji Hospital, Tongji Medical College, Huazhong University of Science and Technology, Wuhan, China; ^2^ Department of Gastroenterology & Endocrinology, Wuhan No. 9 Hospital, Wuhan, China; ^3^ Department of Respiratory Medicine, Tongji Hospital, Tongji Medical College, Huazhong University of Science and Technology, Wuhan, China; ^4^ Department of Gastroenterology, Tongji Hospital, Tongji Medical College, Huazhong University of Science and Technology, Wuhan, China; ^5^ Department of Pediatrics, BC Children’s Hospital Research Institute, University of British Columbia, Vancouver, BC, Canada

**Keywords:** Xpert MTB/RIF, smear microscopy, T-SPOT.*TB*, diagnostic algorithm, real-world study

## Abstract

**Background:**

The prompt diagnosis of pulmonary tuberculosis (PTB) remains a challenge in clinical practice. The present study aimed to optimize an algorithm for rapid diagnosis of PTB in a real-world setting.

**Methods:**

28,171 adult inpatients suspected of having PTB in China were retrospectively analyzed. Bronchoalveolar lavage fluid (BALF) and/or sputum were used for acid-fast bacilli (AFB) smear, Xpert MTB/RIF (Xpert), and culture. A positive mycobacterial culture was used as the reference standard. Peripheral blood mononuclear cells (PBMC) were used for T-SPOT.*TB*. We analyzed specimen types’ effect on these assays’ performance, determined the number of smears for diagnosing PTB, and evaluated the ability of these assays performed alone, or in combination, to diagnose PTB and nontuberculous mycobacteria (NTM) infections.

**Results:**

Sputum and BALF showed moderate to substantial consistency when they were used for AFB smear or Xpert, with a higher positive detection rate by BALF. 3-4 smears had a higher sensitivity than 1-2 smears. Moreover, simultaneous combination of AFB and Xpert correctly identified 44/51 of AFB^+^/Xpert^+^ and 6/7 of AFB^+^/Xpert^-^ cases as PTB and NTM, respectively. Lastly, when combined with AFB/Xpert sequentially, T-SPOT showed limited roles in patients that were either AFB^+^ or Xpert^+^. However, T-SPOT^MDC^ (manufacturer-defined cut-off) showed a high negative predicative value (99.1%) and suboptimal sensitivity (74.4%), and TBAg/PHA (ratio of *Mycobacterium tuberculosis*-specific antigens to phytohaemagglutinin spot-forming cells, which is a modified method calculating T-SPOT.*TB* assay results) ≥0.3 demonstrated a high specificity (95.7%) and a relatively low sensitivity (16.3%) in AFB^-^/Xpert^-^ patients.

**Conclusions:**

Concurrently performing AFB smear (at least 3 smears) and Xpert on sputum and/or BALF could aid in rapid diagnosis of PTB and NTM infections in a real-world high-burden setting. If available, BALF is preferred for both AFB smear and Xpert. Expanding this algorithm, PBMC T-SPOT^MDC^ and TBAg/PHA ratios have a supplementary role for PTB diagnosis in AFB^-^/Xpert^-^ patients (moderately ruling out PTB and ruling in PTB, respectively). Our findings may also inform policy makers’ decisions regarding prevention and control of TB in a high burden setting.

## Introduction

Tuberculosis (TB) caused by the pathogen *Mycobacterium tuberculosis* (*M. tuberculosis*, MTB) continues to pose a major threat to public health. It is estimated that about one quarter of the world’s population is infected with MTB, and 5–10% of those infected will develop TB disease throughout their lifetime (WHO, 2020). While progress has been made in reducing the TB burden worldwide, it has been insufficient to reach the first milestones of the End TB Strategy (WHO, 2018; WHO, 2020). One of the key hurdles to achieving these milestones is the high prevalence of drug resistant TB (Zhao et al., 2012). Moreover, MTB and nontuberculous mycobacteria (NTM) infections often cause indistinguishable clinical symptoms, but their treatment can be vastly different (Forbes et al., 2018).

Rapid and accurate diagnosis of TB is required for effective TB control. Typical TB diagnostic tools include acid-fast bacilli (AFB) smear microscopy, culture, Xpert MTB/RIF (Xpert), and interferon gamma (IFN-γ) releasing assays (IGRAs) (Theron et al., 2012; Forbes et al., 2018). Sputum AFB smear microscopy is the most widely used TB diagnostic test (Forbes et al., 2018). A positive culture of MTB from clinical samples is the gold standard for diagnosing active TB (ATB) infections. However, due to its time-consuming and laborious nature, culture is not often implemented in routine practice. Xpert is a PCR-based test that simultaneously detects MTB and rifampin resistance (Forbes et al., 2018). It is highly sensitive and specific. IGRAs, such as T-SPOT*.TB* [T-SPOT], are T-cell based assays that measure IFN-γ release in response to MTB-specific antigens (Sester et al., 2011) and can yield relatively fast results (usually within one day). IGRAs can be used for diagnosing latent TB infections (LTBI), but cannot be used to rule in or rule out ATB (Mazurek et al., 2010; Sester et al., 2011). Intriguingly, we found that TBAg/PHA ratios (the larger of ESAT-6/PHA and CFP-10/PHA ratios) in the T-SPOT.*TB* assay could be used to distinguish between ATB and LTBI (Wang et al., 2016). Whether TBAg/PHA ratios can be used to diagnose ATB in a real-world setting remains unclear.

There are many different algorithms that integrate the above assays for diagnosing pulmonary TB (PTB). However, this can also complicate health providers’ decisions in choosing optimal PTB diagnostic assays, and sometimes create a “know-do gap” scenario where health providers generally know which algorithms are recommended but in practice use something different (Datta et al., 2017). Moreover, the performance of these algorithms can be affected by the types of specimens (such as sputum vs. BALF), the number of AFB smears and other factors (Conde et al., 2000; Monkongdee et al., 2009). Therefore, it is necessary to identify an optimal algorithm for rapid diagnosis of PTB in a real-world setting.

We retrospectively analyzed a large real-world data set on the diagnosis of PTB. This included assessing the effect of specimen types on the performance of PTB diagnostic assays, determining the number of smears for diagnosing PTB, and evaluating the ability of these assays performed alone, or in combination, to diagnose PTB and NTM infections. Through these rigorous analyses, we were able to identify an optimal algorithm for rapid diagnosis of PTB and NTM infections in a real-world setting.

## Methods

### Study Population

Between January 2016 and March 2019, data from inpatients (≥18 years) undergoing evaluation for PTB (having PTB-related symptoms and/or signs, or unexplained cough lasting ≥2 weeks, or unexplained findings on chest radiographs suggestive of PTB) in Tongji Hospital (Wuhan, China) were included. Tongji hospital is the sixth largest hospital (with 5000 beds) in China, and has been certified by both ISO 15189 (Medical Laboratories-Particular Requirements for Quality and Competence) and CAP (College of American Pathologists).

### Specimen Collection and Processing

Bronchoscopy-derived BALF and expectoration-derived unconcentrated sputum were used for AFB smear, Xpert, and culture tests. About 40 ml of BALF was collected after instilling 30-50 ml of sterile saline (0.9%) into the airway of the affected lung segment. AFB smears and mycobacterial cultures were conducted as previously described (Forbes et al., 2018), but with minor modifications. Briefly, AFB smears on unconcentrated sputum and concentrated BALF (pelleted after centrifugation) were screened using the auramine fluorescence staining method (Baso Diagnostics Inc. Zhuhai, China). Auramine positive AFB smears were also confirmed by Ziehl–Neelsen staining (Baso Diagnostics Inc. Zhuhai, China), a method that appears to have a high specificity for diagnosing ATB (Tarhan et al., 2003; Lee et al., 2018). As for cultures, all sputum and BALF samples were mixed with an equal volume of a 0.5% N-acetyl-l-cysteine-2.0% NaOH and incubated at 37°C for 15-20 min. The mixture was then neutralized by the addition of phosphate buffer (pH 6.8), followed by centrifugation at 3,000 × g for 15 min. After resuspending the pellet in 2 ml of the phosphate buffer, 0.5 ml of the suspension was inoculated into liquid medium (BACTEC 960/MGIT, Becton Dickinson Diagnostic Instrument Systems, Sparks, MD) and 0.2 ml of the suspension was inoculated onto solid medium (Lowenstein-Jensen, Baso Diagnostics Inc. Zhuhai, China). Cultures were grown for 8 weeks. To distinguish between MTB and NTM, positive cultures were tested using the TBAg MPT64 assay (a MPT64-based rapid immunochromatographic kit, GENESIS, Kaibili, China). Cultures negative for TBAg MPT64 were reported as NTM, or subjected to 16S rRNA sequencing to identify the mycobacterial species.

PTB was defined as at least one of the BALF and/or sputum specimens having a positive culture result for *M. tuberculosis* from liquid and/or solid media. A similar approach was used to define active NTM and Nocardia infections.

Xpert was conducted according to the manufacturer’s instructions (Cepheid, Sunnyvale, California). Briefly, untreated sputum samples or BALF samples that were pelleted after centrifugation were mixed with the sample reagent at 1:2 ratio (vol/vol), and incubated at 20–30°C for about 15 min (the mixtures were vortexed for at least 10 seconds between 5 and 10 minutes). About 2 ml of the sample reagent-treated sample was then transferred into the sample chamber of the Xpert cartridge. Xpert results were reported according to the manufacturer’s recommended semi-quantitative classification of the cycle-threshold (Ct) values: high (Ct ≤ 16), medium (16<Ct ≤ 22), low (22<Ct ≤ 28), and very low (Ct>28). If initial Xpert results were non-determinate (error, invalid or no result), testing was repeated with the leftover sample reagent-treated sample (at least 2 ml). In case there was less than 2 ml of sample-reagent-treated sample left, the leftover from the original sample was treated with sample reagent and re-tested as above.

Peripheral blood mononuclear cell (PBMC) T-SPOT*.TB* assay was performed with the T-SPOT ELISpot assay according to the manufacturer’s instructions (Oxford Immunotec Ltd., Oxford, England). Briefly, 2.5 ×10^5^ PBMCs were added to 96-well plates pre-coated with anti-IFN-γ antibody. After incubation for 16–20 h at 37°C with 5% CO2, plates were washed with phosphate buffered saline and developed using an anti-IFN-γ antibody conjugate and substrate, and detected for the presence of secreted IFN-γ. Spot-forming cells (sfc) were counted with an automated ELISpot reader (CTL Analyzers, Cleveland, OH, USA). To report a case of PTB, we used two different methods. One was to use the manufacturer-defined cut-off (T-SPOT^MDC^), and the other was to use ratios of *Mycobacterium tuberculosis*-specific antigens (TBAg) to phytohaemagglutinin (PHA) sfc (TBAg/PHA) as previously described (Wang et al., 2016). Briefly, the ratios of ESAT-6 sfc to PHA sfc and CFP-10 sfc to PHA sfc were calculated, with the larger of the two values representing the TBAg/PHA ratio of one sample.

### Statistical Analysis

AFB smear-positive (AFB^+^) status was based on per-person results (defined as at least one of the BALF and/or sputum specimens having a positive AFB smear), unless otherwise stated. Culture-confirmed PTB and NTM infections were defined as at least one of the BALF and/or sputum specimens having a positive MTB or NTM culture. A positive mycobacterial culture from solid and/or liquid media was used as the reference standard. Comparisons of sensitivities and specificities between independent subgroups of interest were assessed using χ2 test. The *kappa* coefficients were calculated to determine the agreement between BALF and sputum. The agreement of the results (*kappa* value) was categorized as near perfect (0.8–1.0), substantial (0.6–0.8), moderate (0.4–0.6), fair (0.2–0.4), slight (0–0.2), or poor (<0) (Roberts, 2008). All analyses were performed using SPSS version 19 (IBM, Chicago, Illinois), with results considered significantly different at *p*<0.05.

## Results

### Demographic and Clinical Characteristics of Study Population

A total of 28,192 inpatients were screened for eligibility. 21 patients received TB treatment 1 month before hospitalization and were not included (**Supplementary Table S1**). Sputum and/or BALF culture results were available for 7,528 patients, with 8.9% and 1.2% being positive for MTB and NTM, respectively. Among the cultured NTM strains, 25 were identified to species level: 12 *M. avium-intracellulare* complex, 8 *M. fortuitum*, 4 *M.abscessus*, and 1 *M. kansasii*.

### Preferences in Choosing PTB Diagnostic Assays in Real Practice

TB tests ordered by clinicians were variable, including 8,866 AFB, 9,388 AFB/T-SPOT, and many other combinations of tests (**Supplementary Figure S1** and **Table S2**). While AFB and T-SPOT were the first and second most frequently ordered tests, respectively, the percentage of patients undergoing Xpert increased rapidly from 0.8% in 2016 to 17.3% in 2019.

### Consistency Between Sputum and BALF for Diagnosing PTB

In a real-world setting, very few patients had their sputum and BALF collected simultaneously for single PTB diagnostic assay. We determined the consistency between sputum and BALF when they were used for AFB smear, culture, and Xpert. Patients having both sputum and BALF collected within one week of hospitalization for AFB smear (n=3,975), culture (n=109), and Xpert (n=181) analysis were included (**Supplementary Table S3**). Sputum and BALF showed moderate to substantial consistency when used for AFB smear, culture, and Xpert. The positive detection rate by BALF was higher than that by sputum, when they were used for AFB smear or Xpert. The positive detection rate by sputum culture was slightly but insignificantly higher than that by BALF culture.

### Number of Smears to Diagnose PTB

A total of 7,155 patients had 1-8 BALF and/or sputum AFB smears tested within one week of hospitalization (**Supplementary Table S4**). The overall sensitivity of 1-4 AFB smears was 24.6%, 33.4%, 36.2%, and 37.3%, respectively (**Table 1**). While one AFB smear was able to detect 64.7% of AFB^+^ patients with positive MTB culture, two AFB smears increased the detection rate to 88.0% (**Supplementary Table S5**). Three AFB smears detected a further 7.4% of AFB^+^ TB patients as compared to two AFB smears. Four smears detected 98.3% of AFB^+^ TB patients.

**Table 1:** Performance of acid-fast bacilli smears for diagnosing pulmonary tuberculosis.

Accumulated AFB smears (N)	Accumulated samples (N)	Sensitivity % (95% CI)	Positive/total	Specificity% (95% CI)	Negative/total	PPV % (95% CI)	NPV % (95% CI)
**1**	7,155	24.6 (21.2-27.9)	156/635	99.6 (99.4-99.7)	6,492/6,520	84.8 (79.6-90.0)	93.1 (92.5-93.7)
**2**	10,688	33.4 (29.7-37.1)	212/635	99.5 (99.3-99.7)	6,487/6,520	86.5 (82.3-90.8)	93.9 (93.3-94.4)
**3**	12,160	36.2 (32.5-40.0)	230/635	99.5 (99.3-99.6)	6,485/6,520	86.8 (82.7-90.9)	94.1 (93.6-94.7)
**4**	12,993	37.3 (33.6-41.1)	237/635	99.5 (99.3-99.6)	6,484/6,520	86.8 (82.8-90.8)	94.2 (93.7-94.8)
**5**	13,282	37.6 (33.9-41.4)	239/635	99.4 (99.2-99.6)	6,482/6,520	86.3 (82.2-90.3)	94.2 (93.7-94.8)
**6**	13,419	38.0 (34.2-41.7)	241/635	99.4 (99.2-99.6)	6,481/6,520	86.1 (82.1-90.1)	94.3 (93.7-94.8)
**7**	13,488	38.0 (34.2-41.7)	241/635	99.4 (99.2-99.6)	6,481/6,520	86.1 (82.1-90.1)	94.3 (93.7-94.8)
**8**	13,526	38.0 (34.2-41.7)	241/635	99.4 (99.2-99.6)	6,481/6,520	86.1 (82.1-90.1)	94.3 (93.7-94.8)

A total of 7,155 patients, who had 1-8 BALF and/or sputum AFB smears, as well as BALF and/or sputum cultures (single or multiple per person) performed simultaneously during hospitalization, were included in the analysis. Pulmonary tuberculosis was defined as at least one of the BALF and/or sputum specimens having one positive culture result for M. tuberculosis. AFB, acid-fast bacilli; PPV, positive predictive value; NPV, negative predictive value; CI, confidence interval; BALF, bronchoalveolar lavage fluid.

### Performance of AFB Smear, Xpert, or T-SPOT Alone in Diagnosing PTB

A total of 2,044 patients had their respiratory samples tested for AFB smear, culture, Xpert, and T-SPOT (**Table 2**). Both AFB smear and Xpert showed great specificity (>95%), but the sensitivity of AFB smear was much lower than that of Xpert (19.8% vs. 79.7%). Depending on AFB smear status, Xpert performance was different. Xpert was able to identify 97.8% of AFB^+^/culture-positive (culture^+^) TB patients, but only 75.3% of AFB smear-negative (AFB^-^)/culture^+^ TB patients (**Supplementary Table S6**). Despite these findings, Xpert was not performed in 4,252 patients who had both AFB smear and culture results available (**Supplementary Table S7**). Of these patients, 326 (7.7%) were MTB culture^+^, including 212 (65.0%) that were AFB^-^ (**Supplementary Table S8**).

**Table 2:** Performance of acid-fast bacilli smear, Xpert MTB/RIF, and T-SPOT.*TB*, alone or in combination, in diagnosing pulmonary tuberculosis.

Methodology	T-SPOT status	Sensitivity % (95% CI)^†^	Positive/total	Specificity%(95% CI)^‡^	Negative/total	PPV %(95% CI)	NPV %(95% CI)^§^
AFB	.	19.8 (14.7-25.0)^1^	45/227	99.3 (98.9-99.7)^2^	1,804/1,817	77.6 (66.9-88.3)	90.8 (89.6-92.1)^3^
Xpert	.	79.7 (74.5-85.0)	181/227	95.3 (94.3-96.0)	1,731/1,817	67.8 (62.2-73.4)	97.4 (96.7-98.2)
T-SPOT^¶^	T-SPOT^MDC^	81.4 (76.2-86.5)^4^	179/220	69.1 (66.9-71.2)^5^	1,240/1,795	24.4 (21.3-27.5)	96.8 (95.8-97.8)^6^
	TBAg/PHA≥0.3	37.3 (30.9-43.7)^7^	82/220	94.8 (93.7-95.8)^8^	1,701/1,795	46.6 (39.2-54.0)	92.5 (91.3-93.7)^9^
AFB/Xpert	.	80.2 (75.0-85.4)^10^	182/227	94.9 (93.9-95.9)^11^	1,725/1,817	66.4 (60.8-72.1)	97.5 (96.7-98.2)^12^
AFB/T-SPOT^¶^	T-SPOT^MDC^	84.6 (79.8-89.3)^13^	186/220	68.9 (66.8-71.1)^14^	1,237/1,795	25 (21.9-28.1)	97.3 (96.4-98.2)^15^
	TBAg/PHA≥0.3	43.2 (36.6-49.7)^16^	95/220	94.2 (93.1-95.3)^17^	1,691/1,795	47.7 (40.8-54.7)	93.1 (92.0-94.3)^18^
Xpert/T-SPOT^¶^	T-SPOT^MDC^	95 (92.1-97.9)^19^	209/220	67.6 (65.4-69.7)^20^	1,213/1,795	26.4 (23.4-29.5)	99.1 (98.6-99.6)^21^
	TBAg/PHA≥0.3	83.6 (78.8-88.5)^22^	184/220	91.1 (89.8-92.5)^23^	1,636/1,795	53.6 (48.4-58.9)	97.9 (97.2-98.5)^24^
AFB/Xpert/T-SPOT^¶^	T-SPOT^MDC^	95.0 (92.1-97.9)^25^	209/220	67.4 (65.2-69.6)^26^	1,210/1,795	26.3 (23.3-29.4)	99.1 (98.6-99.6)^27^
	TBAg/PHA≥0.3	84.1 (79.3-88.9)^28^	185/220	90.4 (89.1-91.8)^29^	1,623/1,795	51.8 (46.6-57)	97.9 (97.2-98.6)^30^

A total of 2,044 patients had BALF and/or sputum AFB, culture, and Xpert assays, as well as peripheral blood mononuclear cell T-SPOT performed simultaneously. For strict comparison of the performance of AFB, Xpert, and T-SPOT, alone or in combination, only the first AFB, Xpert, and T-SPOT test results were used in the analysis. Pulmonary tuberculosis was defined as at least one of the BALF and/or sputum specimens having one positive culture result for M. tuberculosis. ^¶^Twenty-nine patients with invalid T-SPOT results (PHA spot forming cells <20) were excluded from the analysis, including 7 culture-confirmed MTB cases (1 AFB^+^/Xpert^-^, 4 AFB^-^/Xpert^+^, and 2 AFB^-^/Xpert^-^), 1 NTM cases with AFB^+^/Xpert^-^, and 21 culture-negative cases (1 AFB^-^/Xpert^+^ and 20 AFB^-^/Xpert^-^). PPV=positive predictive value; NPV=negative predictive value; CI=confidence interval; AFB=acid-fast bacilli smear; Xpert=Xpert MTB/RIF; T-SPOT=T-SPOT.TB; T-SPOT^MDC^=manufacturer-defined cutoff; TBAg=Mycobacterium tuberculosis-specific antigen; PHA=phytohaemagglutinin; BALF=bronchoalveolar lavage fluid.†Sensitivity comparison with Xpert: ^1^p<0.0001. ^4^p=0.664. ^7^p=0.0001. ^10^p<0.907. ^19^p<0.0001. ^22^p=0.287. Sensitivity comparison with AFB: ^13^p<0.0001. ^16^p<0.0001. Sensitivity comparison with AFB/Xpert: ^25^p<0.0001. ^28^p=0.280. ^‡^Specificity comparison with Xpert: ^2^p<0.0001. ^5^p<0.0001. ^8^p=0.487. ^11^p=0.645. ^20^p<0.0001. ^23^p<0.0001. Specificity comparison with AFB: ^14^p<0.0001. ^17^p<0.0001. Specificity comparison with AFB/Xpert: ^26^p<0.0001. ^29^p<0.0001. **^§^**NPV comparison with Xpert: ^3^p<0.0001. ^6^p=0.315. ^9^p<0.0001. ^12^p=0.931. ^21^p=0.001. ^24^p=0.401. NPV comparison with AFB: ^15^p<0.0001. ^18^p=0.01. NPV comparison with AFB/Xpert: ^27^p=0.001. ^30^p=0.403.

In addition to AFB smear and Xpert, T-SPOT performance was analyzed. We used two different methods in the T-SPOT assay to define a PTB case, with one method using the manufacturer-defined cut-off (T-SPOT^MDC^), and the other using the TBAg/PHA ratios as previously described (Wang et al., 2016). While T-SPOT^MDC^ and Xpert demonstrated similar sensitivity (**Table 2**), T-SPOT^MDC^ had much lower specificity (69.1%) than Xpert (95.3%). When TBAg/PHA ratios were used, the specificity increased significantly, but at the expense of reduced sensitivity. For instance, TBAg/PHA ≥0.3 demonstrated an overall sensitivity of 37.3% and specificity of 94.8% (**Table 2**). TBAg/PHA ≥0.5 gave an overall sensitivity of 17.3% and specificity of 97.1%. Increasing the TBAg/PHA cut-off to 1.0 decreased the sensitivity to 9.1%, but increased the specificity to 99.0% (**Supplementary Table S9**).

### Use AFB Smear and Xpert to Distinguish Between PTB and NTM Infections

While combining AFB smear and Xpert did not further increase their sensitivity and specificity in diagnosing PTB compared to Xpert alone (**Table 2**), they were able to differentiate PTB and NTM cases more effectively (**Table 3**). The majority (44/51) of AFB^+^/Xpert-positive (Xpert^+^) patients were MTB culture^+^, and the remaining seven patients were culture^-^ but diagnosed as having TB disease based on clinical presentations. Of the 216 AFB^-^/Xpert^+^ patients, 137 and 73 were MTB culture^+^ and culture^-^/clinically active TB, respectively. Six of seven AFB^+^/Xpert^-^ patients were NTM culture^+^. Of 1,770 AFB^-^/Xpert^-^ patients, the majority (1,710) were negative for both MTB and NTM culture. Together, a combination of AFB and Xpert was able to detect 80.2% of patients with culture-proven PTB, and 28.6% of patients with culture-proven NTM.

**Table 3:** Culture results of patients with different acid-fast bacilli smear and Xpert MTB/RIF status.

Culture	AFB^+^/Xpert^+^	AFB^+^/Xpert^-^	AFB^-^/Xpert^+^	AFB^-^/Xpert^-^	Total
**MTB**	44	1	137	45*	227
**NTM**	0	6^†^	0	15^‡^	21
**Nocardia**	0	0	0	3	3
**Negative**	7^†^	0	79**^¶^**	1,707	1,793
**Total**	51	7	216	1,770	2,044

A total of 2,044 patients had BALF and/or sputum AFB smear, culture, Xpert assays, and peripheral blood mononuclear cell T-SPOT performed simultaneously. For strict comparison, only the first AFB smear and Xpert test results were used in the analysis. Culture results were per-patient results (i.e., MTB positivity was defined as at least one of the BALF and/or sputum specimens having one positive culture result for M. tuberculosis. A similar approach was used to define active NTM and Nocardia infections). *None of them were clinically diagnosed as having active tuberculosis. ^†^All of them were clinically diagnosed as having definite or probable tuberculosis. ^‡^None of them were clinically diagnosed as having NTM infections. **^¶^**Six of them had no tuberculosis-related diagnosis. AFB, acid-fast bacilli; AFB^+^, AFB smear positive; AFB^–^, AFB smear negative; Xpert. Xpert MTB/RIF; Xpert^+^, Xpert positive; Xpert^–^, Xpert negative; MTB, M. tuberculosis; NTM, nontuberculous mycobacteria; T-SPOT, T-SPOT.TB; BALF, bronchoalveolar lavage fluid.

### Use T-SPOT in Conjunction With AFB Smear and/or Xpert to Diagnose PTB

We asked if combining T-SPOT with AFB smear and/or Xpert would improve PTB diagnosis. The sensitivity and specificity of AFB/T-SPOT^MDC^ combination was comparable to those of T-SPOT^MDC^ alone, suggesting this combination does not improve PTB diagnosis (**Table 2**). However, when T-SPOT^MDC^ was used together with Xpert, the sensitivity and negative predictive value (NPV) increased to 95.0% and 99.1%, respectively, much higher than those of Xpert or T-SPOT^MDC^ alone (**Table 2**). Adding AFB smear into Xpert/T-SPOT^MDC^ combination did not further increase the sensitivity and NPV. Notably, although combining T-SPOT^MDC^ with Xpert or AFB/Xpert greatly increased sensitivity, it was at the expense of reduced specificity (<67.6%). When TBAg/PHA≥0.3 (**Table 2**) (compared to T-SPOT^MDC^) was used in conjunction with AFB smear and/or Xpert, the specificity increased significantly. These results suggest that TBAg/PHA≥0.3 have some added values for PTB diagnosis when combined with AFB smear and/or Xpert.

### T-SPOT Performance in Diagnosing PTB When Stratified by AFB Smear and Xpert Status

While the above results analyzed the performance of T-SPOT in the overall population, it remained unclear if T-SPOT would perform differently among patients with different AFB smear and Xpert status. We first defined T-SPOT performance based on AFB smear or Xpert results. For AFB^+^ or Xpert^+^ patient populations, T-SPOT^MDC^ showed suboptimal sensitivities (84.1% vs. 83.1%) and very low NPVs (30.0% vs. 47.4%) (**Table 4**). For AFB^-^ or Xpert^-^ patient populations, T-SPOT^MDC^ also showed suboptimal sensitivities (74.4-80.7%), but much higher NPVs (97.3-99.1%). When TBAg/PHA≥0.3 was used, the specificities increased significantly but at the cost of decreased sensitivities (**Table 2**).

**Table 4:** Performance of T-SPOT.*TB* in detecting pulmonary tuberculosis patients with different acid-fast bacilli smear and/or Xpert MTB/RIF status.

AFB smear/Xpert status (N)*	T-SPOT status	Sensitivity % (95% CI)	Positive/total	Specificity% (95% CI)	Negative/total	PPV % (95% CI)	NPV % (95% CI)	Specificity in NTM cases %(n/N)
**AFB^+^** **(56)**	T-SPOT^MDC^	84.1 (73.3-94.9)	37/44	25 (0.5-49.5)	3/12	80.4 (69.0-91.9)	30 (1.6-58.4)	60.0 (3/5)
	TBAg/PHA≥0.3	20.5 (8.5-32.4)	9/44	83.3 (62.3-104.4)	10/12	81.8 (59.0-104.6)	22.2 (10.1-34.4)	100 (5/5)
**AFB^-^** **(1,959)**	T-SPOT^MDC^	80.7 (74.9-86.5)	142/176	69.4 (67.2-71.5)	1,237/1,783	20.6 (17.6-23.7)	97.3 (96.4-98.2)	100 (15/15)
	TBAg/PHA≥0.3	29.0 (22.3-35.7)	51/176	94.8 (93.8-95.9)	1,691/1,783	35.7 (27.8-43.5)	93.1 (92.0-94.3)	100 (15/15)
**Xpert^+^** **(262)**	T-SPOT^MDC^	83.1 (77.5-88.6)	147/177	31.8 (21.9-41.7)	27/85	71.7 (65.5-77.9)	47.4 (34.4-60.3)	No NTM
	TBAg/PHA≥0.3	29.9 (23.2-36.7)	53/177	76.5 (67.5-85.5)	65/85	72.6 (62.4-82.8)	34.4 (27.6-41.2)	No NTM
**Xpert^-^** **(1,753)**	T-SPOT^MDC^	74.4 (61.4-87.5)	32/43	71.0 (68.8-73.1)	1,213/1,710	6.1 (4.0-8.1)	99.1 (98.6-99.6)	90.0 (18/20)
	TBAg/PHA≥0.3	16.3 (5.2-27.3)	7/43	95.7 (94.7-96.6)	1,636/1,710	8.6 (2.5-14.8)	97.9 (97.2-98.5)	100 (20/20)
**AFB^-^/Xpert^-^** **(1,748)**	T-SPOT^MDC^	74.4 (61.4-87.5)	32/43	71.0 (68.8-73.1)	1,210/1,705	6.1 (4.0-8.1)	99.1 (98.6-99.6)	100 (20/20)
	TBAg/PHA≥0.3	16.3 (5.2-27.3)	7/43	95.7 (94.7-96.6)	1,631/1,705	8.6 (2.5-14.8)	97.8 (97.1-98.5)	100 (15/15)
**AFB^+^/Xpert^+^** **(51)**	T-SPOT^MDC^	84.1 (73.3-94.9)	37/44	0.0 (0.0-0.0)	0/7	84.1 (73.3-94.9)	0.0 (0.0-.00)	No NTM
	TBAg/PHA≥0.3	20.5 (8.5-32.4)	9/44	71.4 (38.0-104.9)	5/7	81.8 (59.0-104.6)	12.5 (2.3-22.8)	No NTM
**AFB^+^/Xpert^-†^** **(5)**	T-SPOT^MDC^	N/A	.	60 (17.1-102.9)	3/5	0 (0-0)	100 (100-100)	60 (3/5)
	TBAg/PHA≥0.3	N/A	.	100 (100-100)	5/5	N/A	100 (100-100)	100 (5/5)
**AFB^-^/Xpert^+^** **(211)**	T-SPOT^MDC^	82.7 (76.3-89.1)	110/133	34.6 (24.1-45.2)	27/78	68.3 (61.1-75.5)	54 (40.2-67.8)	No NTM
	TBAg/PHA≥0.3	33.1 (25.1-41.1)	44/133	59.1 (44.6-73.6)	26/44	71.0 (59.7-82.3)	22.6 (15.0-30.3)	No NTM

A total of 2,044 patients had BALF and/or sputum AFB smear, culture, and Xpert assays, as well as peripheral blood mononuclear cell T-SPOT performed concurrently. For strict comparison of the performance of T-SPOT in patients with different AFB smear and Xpert status, only the first AFB smear, Xpert, and T-SPOT results were used in the analysis. Twenty-nine patients with invalid T-SPOT results (PHA spot forming cells <20) were excluded from the analysis, including 7 culture-confirmed MTB cases (1 AFB^+^/Xpert^-^, 4 AFB^-^/Xpert^+^, and 2 AFB^-^/Xpert^-^), 1 NTM cases with AFB^+^/Xpert^-^, and 21 culture-negative cases (1 AFB^-^/Xpert^+^ and 20 AFB^-^/Xpert^-^). Pulmonary tuberculosis was defined as at least one of the BALF and/or sputum specimens having one positive culture result for M. tuberculosis. *Number of patients with different AFB smear and Xpert status. ^†^No tuberculosis cases. AFB, acid-fast bacilli; AFB^+^, AFB smear positive; AFB^–^, AFB smear negative; Xpert, Xpert MTB/RIF; Xpert^+^, Xpert positive; Xpert^–^, Xpert negative; PPV, positive predictive value; NPV, negative predictive value; NTM, nontuberculous mycobacteria; CI, confidence interval; N/A, not applicable; T-SPOT, T-SPOT.TB; BALF, bronchoalveolar lavage fluid; MTB, M. tuberculosis.

We then defined T-SPOT performance based on the status of both AFB smear and Xpert. Accordingly, patients were grouped into four populations: AFB^-^/Xpert^-^, AFB^+^/Xpert^+^, AFB^+^/Xpert^-^, and AFB^-^/Xpert^+^ (**Table 4**). For AFB^-^/Xpert^-^ patients, T-SPOT^MDC^ demonstrated a high NPV (99.1%) and a suboptimal sensitivity (74.4%) and specificity (71.0%). When TBAg/PHA≥0.3 was used, the specificity was significantly increased to 95.7% but with a decreased sensitivity (16.3%). In contrast, T-SPOT^MDC^ and TBAg/PHA showed no added values in 51 AFB^+^/Xpert^+^ patients and 211 AFB^-^/Xpert^+^ patients who were either MTB culture^+^ or clinically diagnosed as having PTB. T-SPOT performance was inconclusive in AFB^+^/Xpert^-^ patients (n=5), although it ruled out PTB in three NTM culture^+^ cases.

## Discussion

While there are many meta-analyses and pro/retrospective studies addressing the performance of individual TB tests (AFB smear, Xpert, and T-SPOT), very few studies compared the performance of these tests in a holistic view in a real-world setting. Moreover, there are no real-world studies deciphering how these individual tests should be integrated into an optimal algorithm for rapid diagnosis of PTB.

**Figure 1:**
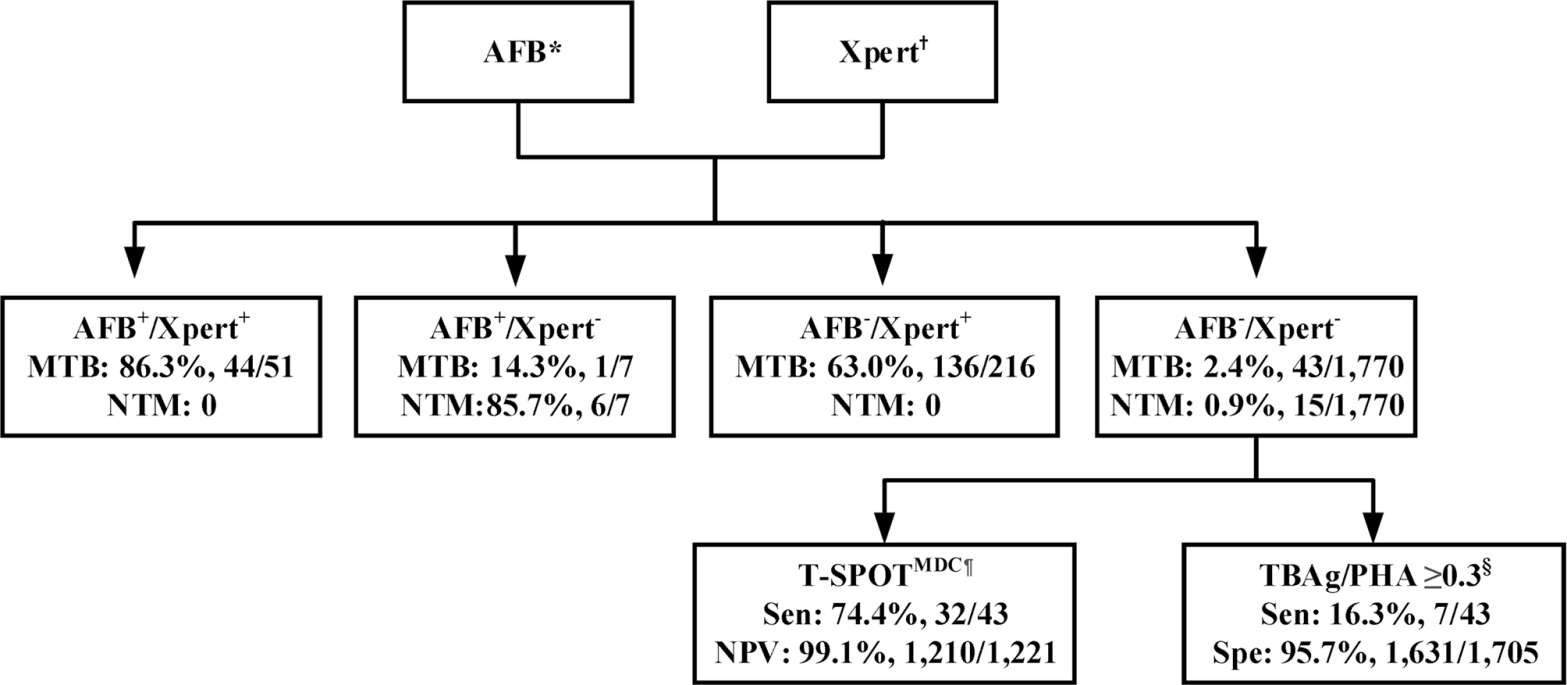
Recommended algorithm for accurate and rapid diagnosis of pulmonary tuberculosis in a real-world setting with high prevalence of *M. tuberculosis* and nontuberculous mycobacterium infections. *Three to four respiratory samples are recommended for AFB smear microscopy, with bronchoalveolar lavage liquid (BALF) preferred. ^†^BALF preferred. **^¶^**T-SPOT^MDC^ (manufacturer-defined cutoff) has a supplementary role in ruling out pulmonary tuberculosis among AFB^-^/Xpert^-^ patients. ^§^TBAg/PHA (ratio of TBAg to PHA spot-forming cells, which is modified method calculating T-SPOT.*TB* assay results) ≥0.3 has a supplementary role in ruling in pulmonary tuberculosis among AFB^-^/Xpert^-^ patients. AFB, acid-fast bacilli smear; AFB^+^, AFB smear positive; AFB^-^, AFB smear negative; Xpert, Xpert MTB/RIF; Xpert^+^, Xpert positive; Xpert^-^, Xpert negative; MTB, *Mycobacterium tuberculosis*; NTM, nontuberculous mycobacterium; T_SPOT, T-SPOT.*TB*; MDC, manufacturer-defined cutoff; TBAg, *Mycobacterium tuberculosis*-specific antigens; PHA, phytohaemagglutinin; Sen, sensitivity; PPV, positive predictive value; Spe, specificity.

To identify such a potential algorithm, we retrospectively analyzed a large real-world data set from a tertiary referral hospital. We found a much higher sensitivity of 3-4 AFB smears compared to 1-2 AFB smears. We also demonstrated the superiority of BALF to sputum for both AFB smear and Xpert, the higher sensitivity of Xpert compared to AFB smear, as well as the significantly improved accuracy of combining Xpert and AFB smear to diagnose MTB and NTM infections. Lastly, we showed that T-SPOT^MDC^ and TBAg/PHA ratios have a supplementary role for PTB diagnosis in AFB^-^/Xpert^-^ patients. These findings led us to propose an optimal algorithm, whereby AFB smear (≥3 smears) and Xpert should be performed first on sputum and/or BALF for rapid diagnosis of MTB and NTM infections in a high-burden setting (**Figure 1**). If available, BALF is preferred for both AFB smear and Xpert. T-SPOT^MDC^ and TBAg/PHA ratios may be useful for diagnosing PTB in AFB^-^/Xpert^-^ patients (moderately ruling out PTB and ruling in PTB, respectively).

Our recommendation that 3-4 AFB smears should be performed is based on two observations: (1) 3-4 smears showed high sensitivities and were capable of identifying >95% of AFB^+^/culture^+^ TB patients; and (2) the quality of respiratory samples in real practice may not be always ideal. Similar to our study, a US algorithm recommended three consecutive sputum smears for AFB staining (Jensen et al., 2005). In contrast, WHO and European Union recommended two consecutive sputum smears in settings with appropriate external quality assurance and high-quality microscopy (Migliori et al., 2018).

The higher sensitivity of Xpert (compared to AFB smear) and lower specificity of T-SPOT^MDC^ (compared to AFB smear and Xpert) for detecting PTB in this study are consistent with those reported by other prospective/retrospective studies (Ling et al., 2011; Metcalfe et al., 2011; Theron et al., 2011; Lee et al., 2013). While this may not be unexpected, it suggests that Xpert is the preferred assay in real practice. Moreover, when Xpert was used in combination with AFB smear, it significantly improved the diagnostic accuracy for PTB and NTM infections. These findings are consistent with the recommendation by US CDC that participants with AFB^+^/nucleic acid amplification test (NAAT) positive and AFB^+^/NAAT-negative respiratory samples are presumable ATB and NTM cases, respectively (Forbes et al., 2018).

Our real-world data also showed that T-SPOT^MDC^ or TBAg/PHA ratio alone was unable to rule in or rule out PTB. When combined with AFB smear or Xpert, they also did not improve the performance compared to AFB smear or Xpert alone. This agrees with findings from other studies (Ling et al., 2011; Metcalfe et al., 2011; Forbes et al., 2018), and supports the WHO policy that IGRAs should not be used for diagnosing active TB (Sester et al., 2011). However, upon stratifying the results of AFB smear and Xpert, T-SPOT^MDC^ and TBAg/PHA ratios showed added values in AFB^-^/Xpert^-^ patients (moderately ruling out and ruling in PTB, respectively), but not in AFB^+^ or Xpert^+^ patients. Similarly, IGRAs showed a moderate performance in ruling out ATB in Xpert^-^ individuals in a high-TB/HIV burden setting (Theron et al., 2012). Intriguingly, a recent study showed that T-SPOT with BALF with a cut-off of >4000 early secretory antigenic target-6- or culture filtrate protein-10-specific interferon-γ-producing lymphocytes per 10^7^ lymphocytes was able to identify 88.9% of AFB^-^/Xpert^-^ patients with culture-proven MTB (Jafari et al., 2018), although the sample size of this study is small. It will be interesting to determine if BALF-based T-SPOT^MDC^ and TBAg/PHA ratios can better predict TB disease within a large AFB^-^/Xpert^-^ population.

Although T-SPOT^MDC^ or the TBAg/PHA ratio alone was unable to rule in or rule out PTB, the TBAg/PHA ratio (≥0.3) showed increased specificity (albeit at the cost of decreased sensitivity) for diagnosing PTB as compared to T-SPOT^MDC^ (**Table 2**). Traditional T-SPOT^MDC^ measures IFN-γ release in response to MTB-specific antigens, but its performance can be greatly affected by host immune status. Interestingly, we found reduced IFN-γ release in response to PHA in active TB (Wang et al., 2016), although the mechanism underlying this remains unclear. By normalizing TBAg IFN-γ release against PHA IFN-γ release (*i.e.* TBAg/PHA ratio), the impact of host immune status appears to be minimized. In fact, this TBAg/PHA ratio was able to outperform T-SPOT^MDC^ in differentiating between ATB and LTBI (Wang et al., 2016).

Thus, our analyses not only validated the performance of individual tests in a real-world setting, but also provided the basis of integrating these tests in a single algorithm to diagnose PTB and NTM infections. Prior to this study, no formal evidence-based PTB diagnostic algorithms have been developed in a real-world setting. As a result, clinicians from this study tended to have different decisions in choosing TB tests. For instance, only 26.7% of patients underwent culture tests (**Supplementary Table S2**), probably reflecting the fact that clinicians prefer to order TB assays with fast turnaround time (such as AFB smear). Indeed, we noticed about 1/3 patients were ordered for AFB smear alone, and another 1/3 of patients were ordered for AFB/T-SPOT. Less than 1/5 of patients were ordered for AFB/Xpert.

Our study has several strengths. All data were collected from a large heterogeneous population, allowing the generation of real-world evidence that confirms findings from studies with selected populations. Furthermore, our diagnostic algorithm included both PTB and NTM infections. A few prospective/retrospective studies have demonstrated improved accuracy of combining AFB smear and PCR-based tests for diagnosing PTB (Tueller et al., 2005; Roberts, 2008; Pan et al., 2018), but did not include NTM diagnosis in their algorithms. Lastly, this algorithm recommends T-SPOT assay only for AFB^-^/Xpert^-^ patients. Benefiting from this algorithm, AFB^+^ or Xpert^+^ patients will not have to undergo T-SPOT assay or pay additional costs.

Our study also has some limitations. We did not include children, for whom PTB diagnosis is more challenging. We also did not evaluate the performance of diagnostic tests in patients with different immune status, such as those co-infected with HIV or having diabetes. This is largely due to insufficient numbers of these patients in a very heterogeneous population. The sample size of NTM infections in this study is still too small. Additionally, fast tests for drug resistance (such as the line probe assay GenoType MTBDRplus) should be incorporated into the algorithm in the future study.

In summary, extensive analyses of a large real-world data set allowed us to identify an optimal algorithm for fast diagnosis of PTB and NTM infections in a high-burden setting (such as China, and probably other lower middle-income countries with a similar situation). Findings from this study may also inform policy makers’ decisions regarding prevention and control of TB at a local and national level. Nevertheless, our future work will be to validate the proposed algorithm through multi-center prospective studies and analyze its cost-effectiveness.

## Data Availability Statement

The raw data supporting the conclusions of this article will be made available by the authors, without undue reservation.

## Ethics Statement

The studies involving human participants were reviewed and approved by Tongji Medical College, Huazhong University of Science & Technology, Wuhan, China. Written informed consent for participation was not required for this study in accordance with the national legislation and the institutional requirements.

## Author Contributions

JP, JS, ZS, and HY conceived and designed the study. JP, FW, WL, YL, FW, LT, ZC, YZ, and TL performed the experiments. JP, JS, XiW, NS, XuW, SW, QY, BAV, KJ, ZS, and HBY interpreted the data. ZS contributed reagents and materials. ZS and HBY supervised this study. JP and HBY wrote the manuscript. All authors contributed to the article and approved the submitted version.

## Funding

This work was supported in part by grants from National Mega Project on Major Infectious Disease Prevention (grant no. 2017ZX10103005-007-001, 2017ZX10103005-007-002).

## Conflict of Interest

The authors declare that the research was conducted in the absence of any commercial or financial relationships that could be construed as a potential conflict of interest.
