# Supplementary material for: Harnessing Big Data to Optimize an Algorithm for Rapid Diagnosis of Pulmonary Tuberculosis in a Real-World Setting
Source: Front Cell Infect Microbiol. 2021 Mar 18;11:650163. doi: 10.3389/fcimb.2021.650163 (PMC8012509; doi:10.3389/fcimb.2021.650163)
Supplement: Supplementary file 1 [file DataSheet_1.pdf]

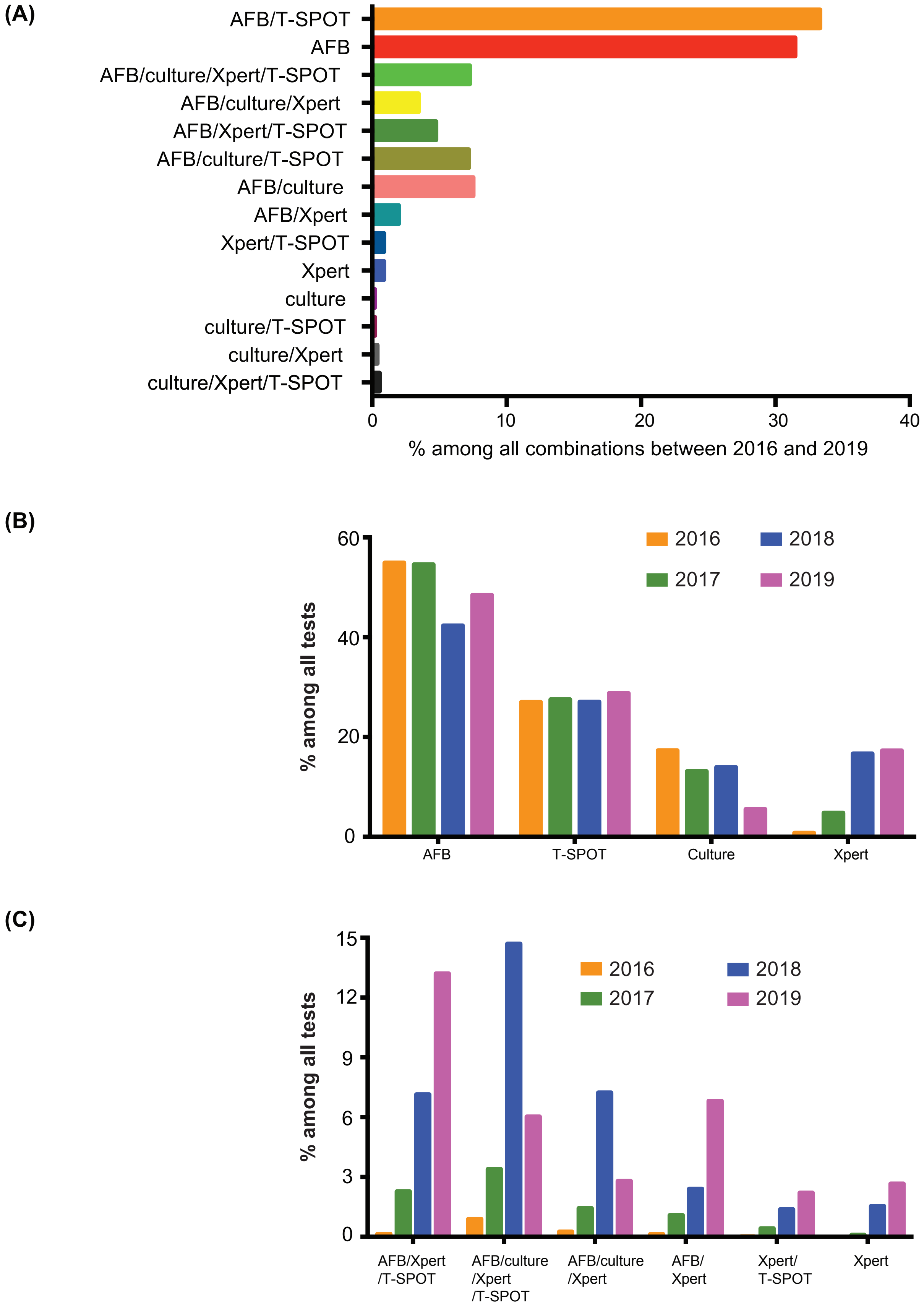

Supplementary Figure S1. Clinicians' preferences in choosing TB diagnositic assays. (A) The overall percentage of patients undergoing single assay or a combination of assays (based on per-patient record) between 2016 and 2019. A total of 28,171 patients were included in the analysis. About one third of patients had AFB smear only or AFB/T-SPOT, followed by AFB/culture (7.5%), AFB/culture/Xpert/T-SPOT (7.3%), and AFB/culture/T-SPOT (7.2%). (B) The percentage of patients undergoing AFB smear, T-SPOT, culture, or Xpert (based on per-patient record) per year between 2016 and 2019. If a patient had a combination of assays, each assay was recorded as from one patient. AFB and T-SPOT were the first and second most frequently ordered tests by clinicians, respectively. (C) The percentage of patients undergoing Xpert containing assay(s) per year between 2016 and 2019. AFB=acid-fast bacillus; T-SPOT=T-SPOT.*TB*; Xpert=Xpert MTB/RIF.
