# Supplementary material for: Harnessing Big Data to Optimize an Algorithm for Rapid Diagnosis of Pulmonary Tuberculosis in a Real-World Setting
Source: Front Cell Infect Microbiol. 2021 Mar 18;11:650163. doi: 10.3389/fcimb.2021.650163 (PMC8012509; doi:10.3389/fcimb.2021.650163)
Supplement: Supplementary file 2 [file DataSheet_2.docx]

| **SUPPLEMENTARY TABLE S1. Demographics and clinical characteristics of subjects included** | | | | | |
| --- | --- | --- | --- | --- | --- |
|  | **Culture-confirmed MTB (n=672)** | **Culture-confirmed NTM*** **(n=89)** | **Culture-negative subjects (n=6,767)** | **Subjects without culture (n=20,643)** | **Total (n=28,171)** |
| **Age, years** |  |  |  |  |  |
| **Mean ± SD** | 48.7±16.1 | 54.6±13.0 | 52.5±13.4 | 57.2±15.6 | 55.9±15.3 |
| **Median (IQR)** | 51 (36-62) | 54 (48-64) | 54 (45-62) | 59 (48-68) | 57 (47-66) |
| **Range** | 18-88 | 20-84 | 18-92 | 18-99 | 18-99 |
| **Male** | 368 (54.8%) | 34 (38.2%) | 3,736 (55.2%) | 12,681 (61.4%) | 16,819 (59.7%) |
| **Female** | 304 (45.3%) | 55 (61.8%) | 3,031 (44.8%) | 7,962 (38.6%) | 11,352 (40.3%) |
| **Other pre-existing conditions or comorbidities^†^** |  |  |  |  |  |
| **None** | 100 (14.9%) | 2 (2.2%) | 157 (2.3%) | 235 (1.1%) | 494 (1.8%) |
| **HIV infected^‡^** | 4 (0.6%) | 0 (0.0%) | 4 (0.1%) | 45 (0.2%) | 53 (0.2%) |
| **Diabetes** | 40 (6.0%) | 0 (0.0%) | 261 (3.9%) | 1,339 (6.5%) | 1,640 (5.8%) |
| **Chronic or end-stage kidney disease** | 0 (0.0%) | 0 (0.0%) | 7 (0.1%) | 69 (0.3%) | 76 (0.3%) |
| **Transplant** | 4 (0.6%) | 0 (0.0%) | 22 (0.3%) | 98 (0.5%) | 124 (0.4%) |
| **Silicosis** | 1 (0.1%) | 1 (1.1%) | 57 (0.8%) | 131 (0.6%) | 190 (0.7%) |
| **Connective tissue disease** | 3 (0.4%) | 2 (2.2%) | 72 (1.1%) | 553 (2.7%) | 630 (2.2%) |
| **Cancer** | 33 (4.9%) | 10 (11.2%) | 704 (10.4%) | 2,069 (10.0%) | 2,816 (10.0%) |
| **Psoriasis** | 0 (0.0%) | 0 (0.0%) | 2 (0.0%) | 6 (0.0%) | 8 (0.0%) |
| **Crohn’s disease** | 0 (0.0%) | 0 (0.0%) | 0 (0.0%) | 5 (0.0%) | 5 (0.0%) |
| **Asthma** | 6 (0.9%) | 0 (0.0%) | 239 (3.5%) | 943 (4.6%) | 1,188 (4.2%) |
| **Hepatitis B** | 2 (0.3%) | 0 (0.0%) | 8 (0.1%) | 95 (0.5%) | 105 (0.4%) |
| **Hepatitis C** | 0 (0.0%) | 0 (0.0%) | 2 (0.0%) | 13 (0.1%) | 15 (0.1%) |
| **Others** | 481 (71.4%) | 71 (79.8%) | 5,342 (78.9%) | 15,553 (75.3%) | 21,446 (76.1%) |
| Data are presented as n (%), unless otherwise stated. Culture results obtained using multiple samples were per-person results (negative: cultures showing no bacterial growth). *Among the cultured NTM strains, 25 were identified to species level: 12 *M. avium-intracellulare c*omplex, 8 *M. fortuitum*, 4 *M.abscessus*, and 1 *M. kansasii*. ^†^Some patients had multiple comorbidities. ^‡^Four TB patients (0.6%, 4/672) were HIV positive, accounting for 50% of HIV-infected patients who had culture results available. While none of the NTM^+^ patients received the culture result (average length from culture to reporting NTM species: 18 days) on discharge, 12 of them were empirically treated with anti-TB drugs during hospitalization. MTB=*Mycobacterium tuberculosis*; NTM= nontuberculous bacterium; SD=standard deviation; IQR=interquartile range; HIV=human immunodeficiency virus; TB=tuberculosis. | | | | | |

| **SUPPLEMENTARY TABLE S2. Clinicians’ preferences in choosing single assay or a combination of assays for diagnosing pulmonary tuberculosis** | |
| --- | --- |
| **Assay (s) used for diagnosing pulmonary tuberculosis** | **Number of patients (%)** |
| **AFB** | 8,866 (31.5) |
| **AFB/T-SPOT** | 9,388 (33.3) |
| **AFB/Culture** | 2,121 (7.5) |
| **AFB/Culture/T-SPOT** | 2,021 (7.2) |
| **AFB/Xpert/T-SPOT** | 1,340 (4.8) |
| **AFB/Culture/Xpert/T-SPOT** | 2,044 (7.3) |
| **AFB/Culture/Xpert** | 969 (3.4) |
| **AFB/Xpert** | 555 (2.0) |
| **Xpert/T-SPOT** | 246 (0.9) |
| **Xpert** | 248 (0.9) |
| **Culture** | 53 (0.2) |
| **Culture/T-SPOT** | 57 (0.2) |
| **Culture/Xpert** | 107 (0.4) |
| **Culture/Xpert/T-SPOT** | 156 (0.6) |
| **Total** | 28,171 (100) |
| The number of patients undergoing single assay or a combination of assays (based on per-patient record) between 2016 and 2019. A total of 28,171 patients were included in the analysis. AFB=acid-fast bacilli smear; T-SPOT=T-SPOT*.TB*; Xpert=Xpert MTB/RIF. | |

| **SUPPLEMENTARY TABLE S3. Impact of specimen types on the performance of acid-fast bacilli smear, culture, and Xpert MTB/RIF** | | | | | | | | | | | | | | | |
| --- | --- | --- | --- | --- | --- | --- | --- | --- | --- | --- | --- | --- | --- | --- | --- |
|  |  | **AFB smear (n=3,975)** | | | |  | **Culture (n=109)** | | | |  | **Xpert（n=181）** | | | |
|  |  | Sputum | | *kappa*  value | *p* value |  | Sputum | | *kappa*  value | *p* value |  | Sputum | | *kappa*  value | *p* value |
|  |  | + | - |  |  |  | + | - |  |  |  | + | - |  |  |
| **BALF** | + | 36 | 68 | 0.406 | <0.0001 |  | 7 | 3 | 0.506 | <0.0001 |  | 17 | 12 | 0.648 | <0.0001 |
|  | - | 32 | 3,839 |  |  |  | 8 | 91 |  |  |  | 3 | 149 |  |  |
| Patients who had both BALF and sputum specimens tested with the same assay were included in this analysis. 3,975, 109, and 181 patients underwent AFB smear, culture, and Xpert, respectively. The Kappa statistic was used to evaluate the agreement of results between BALF and sputum for each assay. The agreement of the results (presented as *kappa* value) was categorized as near perfect (0.8-1.0), substantial (0.6-0.8), moderate (0.4-0.6), fair (0.2-0.4), slight (0-0.2), or poor (<0). McNemar’s test was used to compare the positive detection rates by BALF and sputum. BALF showed a higher positive detection rate compared to sputum, when they were used for AFB smear (2.6% vs. 1.7%, *p*<0.0001), or Xpert (16.0% vs. 11.0%, *p*=0.04). The positive detection rate by sputum culture was 13.8%, slightly but insignificantly (*p*=0.23) higher than that by BALF culture (9.2%). AFB=acid-fast bacilli; Xpert=Xpert MTB/RIF; BALF=bronchoalveolar lavage fluid. | | | | | | | | | | | | | | | |

| **SUPPLEMENTARY TABLE S4. Distribution of patients with single or multiple samples for acid-fast bacilli smear microscopy** | | |
| --- | --- | --- |
| **Number of samples per patient** | **Number of patients (%)** | **Number of samples per category** |
| **1 sample** | 3,622 (50.6) | 3,622 |
| **2 samples** | 2,061 (28.8) | 4,122 |
| **3 samples** | 639 (8.9) | 1,917 |
| **4 samples** | 544 (7.6) | 2,176 |
| **5 samples** | 152 (2.1) | 760 |
| **6 samples** | 68 (1.0) | 408 |
| **7 samples** | 31 (0.4) | 217 |
| **8 samples** | 38 (0.5) | 304 |
| A total of 7,155 patients, who had bronchoalveolar lavage fluid and/or sputum acid-fast bacilli smear and culture performed simultaneously, were included in the analysis. Data are presented as n (%), unless otherwise stated. | | |

| **SUPPLEMENTARY TABLE S5. Performance of various numbers of acid-fast bacilli smears in diagnosing pulmonary tuberculosis among acid-fast bacilli smear-positive patients** | | | | | | |
| --- | --- | --- | --- | --- | --- | --- |
| **Accumulated AFB smears** | **AFB smear status** | **Culture** | | | | |
|  |  | **MTB** | **NTM** | **Nocardia** | **Negative** | **Total** |
| **1** | Positive | 156 (64.7) | 13 (65) | 1 (100) | 14 (77.8) | 184 (65.7) |
| **2** | Positive | 212 (88.0) | 15 (75) | 1 (100) | 17 (94.4) | 245 (87.5) |
| **3** | Positive | 230 (95.4) | 16 (80) | 1 (100) | 18 (100) | 265 (94.6) |
| **4** | Positive | 237 (98.3) | 17 (85) | 1 (100) | 18 (100) | 273 (97.5) |
| **5** | Positive | 239 (99.2) | 19 (95) | 1 (100) | 18 (100) | 277 (98.9) |
| **6** | Positive | 241 (100) | 20 (100) | 1 (100) | 18 (100) | 280 (100) |
| **7** | Positive | 241 (100) | 20 (100) | 1 (100) | 18 (100) | 280 (100) |
| **8** | Positive | 241 (100) | 20 (100) | 1 (100) | 18 (100) | 280 (100) |
| A total of 280 patients, who had 1-8 BALF and/or sputum AFB smear, as well as BALF and/or sputum cultures (single or multiple, per person) performed simultaneously during hospitalization, were included in the analysis. These patients were AFB smear positive. Pulmonary tuberculosis was defined as at least one of the BALF and/or sputum specimens having one positive culture result for MTB. A similar approach was used to define active NTM and Nocardia infections. Data are presented as n (%). AFB=acid-fast bacilli; MTB=*M. tuberculosis*; NTM=nontuberculous mycobacteria; BALF=bronchoalveolar lavage fluid. | | | | | | |

| **SUPPLEMENTARY TABLE S6. Performance of Xpert MTB/RIF in diagnosing pulmonary tuberculosis according to acid-fast bacilli smear status** | | | | | | | | | | | |
| --- | --- | --- | --- | --- | --- | --- | --- | --- | --- | --- | --- |
| **AFB smear status** | **Sensitivity** | |  | **Specificity** | |  | **PPV** | |  | **NPV** | |
|  | **n/N** | **Estimate % (95% CI)** |  | **n/N** | **Estimate %**  **(95% CI)** |  | **n/N** | **Estimate %**  **(95% CI)** |  | **n/N** | **Estimate % (95% CI)** |
| **All** | 181/227 | 79.7  (74.5-85.0) |  | 1731/1817 | 95.3  (94.3-96.2) |  | 181/267 | 67.8  (62.2-73.4) |  | 1731/1777 | 97.4  (96.7-98.2) |
| **AFB^+^** | 44/45 | 97.8 (93.5-102.1) |  | 6/13 | 46.2 (19.1-73.3) |  | 44/51 | 86.3 (76.8-95.7) |  | 6/7 | 85.7 (59.8-111.6) |
| **AFB^-^** | 137/182 | 75.3  (69.0-81.5) |  | 1725/1804 | 95.6  (94.7-96.6) |  | 137/216 | 63.4  (57.0-69.8) |  | 1725/1770 | 97.5  (96.7-98.2) |
| A total of 2,044 patients who had BALF and/or sputum AFB smear, culture, Xpert assays, and peripheral blood mononuclear cell T-SPOT performed concurrently were included in this analysis. For strict comparison, only the first AFB and Xpert test results were used in the analysis. Pulmonary tuberculosis was defined as at least one of the BALF and/or sputum specimens having one positive culture result for *M. tuberculosis*. PPV=positive predictive value; NPV=negative predictive value; CI=confidence interval; AFB=acid-fast bacilli; AFB^+^=AFB smear positive; AFB^–^= AFB smear negative; BALF=bronchoalveolar lavage fluid; Xpert=Xpert MTB/RIF; T-SPOT=T-SPOT.*TB*. | | | | | | | | | | | |

| **SUPPLEMENTARY TABLE S7. Results of patients having acid-fast bacilli smear and culture but no Xpert MTB/RIF** | | | | | |
| --- | --- | --- | --- | --- | --- |
| **AFB smear status** | **Culture** | | | | |
|  | **MTB** | **NTM** | **Nocardia** | **Negative** | **Total** |
| **AFB^+^** | 114 | 7 | 0 | 4 | 125 |
| **AFB^-^** | 212 | 48 | 5 | 3,862 | 4,128 |
| **Total** | 326 | 55 | 5 | 3,866 | 4,252 |
| A total of 4,252 patients had both AFB smear and culture, but did not have Xpert test. All AFB smear and culture results were per-patient results (*i.e.*, MTB positivity was defined as at least one of the BALF and/or sputum specimens having one positive culture result for MTB; a similar approach was used to define active NTM and Nocardia infections). MTB=*M. tuberculosis;* NTM=nontuberculous mycobacteria; AFB=acid-fast bacilli; AFB^+^=AFB smear positive. AFB^–^=AFB smear negative; BALF=bronchoalveolar lavage fluid. | | | | | |

| **SUPPLEMENTARY TABLE S8. Clinical information of patients who were acid-fast bacilli smear-negative but culture-positive for *M. tuberculosis*** | | | | | |
| --- | --- | --- | --- | --- | --- |
| **Diagnosis on discharge*** | **No.** | **Imaging/ pathology** | **TB history** | **Current treatment for TB^†^** | **Referring to TB specialized hospitals^†^** |
| **Definite pulmonary TB** | 23 | 6 | 5 | 13 | 6 |
| **Probable pulmonary TB** | 32 | 22 | 2 | 19 | 5 |
| **Clinically indeterminate TB** | 63 | 10 | 2 | 1 | 2 |
| **Obsolete pulmonary TB** | 6 | 2 | 1 | 0 | 0 |
| **Definite tuberculous pleurisy** | 1 | 1 | 2 | 1 | 0 |
| **Probable tuberculous pleurisy** | 2 | 0 | 0 | 1 | 0 |
| **Clinically indeterminate tuberculous pleurisy** | 1 | 0 | 0 | 1 | 0 |
| **Clinically indeterminate intestinal TB** | 1 | 1 | 0 | 0 | 0 |
| **Clinically indeterminate NTM** | 1 | 0 | 0 | 0 | 0 |
| **Non-TB** | 82 | 14 | 1 | 3 | 1 |
| A total of 212 patients had AFB smear and culture, but no Xpert assays. They were AFB smear-negative but culture-positive for *M. tuberculosis*. The average length of hospitalization for these 212 patients was 9.6 days, and 208 of them showed positive culture results after more than 14 days. On discharge, only seven patients (3.3%, 7/212) received the culture-confirmed TB-positive result. 39 (18.4%, 39/212) received anti-TB treatment during hospitalization, and 14 (6.6%, 14/212) were transferred to TB specialized hospitals for further treatment. *TB related diagnoses were made by clinicians, which were based on clinical symptoms and signs, imaging (chest radiograph, computed tomography)/pathology findings, and/or response to anti-TB treatment. †Ten patients received anti-TB treatment during their hospitalization and were then transferred to TB specialized hospitals; other patients either received treatment for TB or were directly transferred to TB specialized hospitals. TB=tuberculosis; AFB=acid-fast bacilli; Xpert=Xpert MTB/RIF; NTM=nontuberculous mycobacteria. | | | | | |

| **SUPPLEMENTARY TABLE S9. Performance of acid-fast bacilli smear, Xpert MTB/RIF, and T-SPOT.TB, alone or in combination, in diagnosing pulmonary tuberculosis** | | | | | | | |
| --- | --- | --- | --- | --- | --- | --- | --- |
| **Methodology** | **T-SPOT status** | **Sensitivity % (95% CI) ^†^** | **Positive/total** | **Specificity%**  **(95% CI) ^‡^** | **Negative/**  **total** | **PPV %**  **(95% CI)** | **NPV %**  **(95% CI) ^§^** |
| AFB | .. | 19.8  (14.7-25.0)^1^ | 45/227 | 99.3  (98.9-99.7)^2^ | 1,804/1,817 | 77.6  (66.9-88.3) | 90.8  (89.6-92.1)^3^ |
| Xpert | .. | 79.7  (74.5-85.0) | 181/227 | 95.3  (94.3-96.0) | 1,731/1,817 | 67.8  (62.2-73.4) | 97.4  (96.7-98.2) |
| T-SPOT^¶^ | TBAg/PHA≥0.5 | 17.3  (12.3-22.3)^4^ | 38/220 | 97.1  (96.3-97.8)^5^ | 1,742/1,795 | 41.8  (31.6-51.9) | 90.5  (89.2-91.9)^6^ |
|  | TBAg/PHA≥1.0 | 9.1  (5.3-12.9)^7^ | 20/220 | 99.0  (98.5-99.5)^8^ | 1,778/1,796 | 52.6  (36.8-68.5) | 89.9  (88.6-91.2)^9^ |
| AFB/Xpert | .. | 80.2  (75.0-85.4)^10^ | 182/227 | 94.9  (93.9-95.9)^11^ | 1,725/1,817 | 66.4  (60.8-72.1) | 97.5  (96.7-98.2)^12^ |
| AFB/T-SPOT^¶^ | TBAg/PHA≥0.5 | 34.1  (27.8-40.4)^13^ | 75/220 | 96.5  (95.6-97.3)^14^ | 1,732/1,795 | 54.4  (46.0-62.7) | 92.3  (91.1-93.5)^15^ |
|  | TBAg/PHA≥1.0 | 27.3  (21.4-33.2)^16^ | 60/220 | 98.5  (97.9-99.1)^17^ | 1,768/1,795 | 69.0  (59.2-78.7) | 91.7  (90.5-92.9)^18^ |
| Xpert/T-SPOT^¶^ | TBAg/PHA≥0.5 | 83.2  (78.2-88.1)^19^ | 183/220 | 93.0  (91.8-94.2)^20^ | 1,669/1,795 | 59.2  (53.7-64.7) | 97.8  (97.1-98.5)^21^ |
|  | TBAg/PHA≥1.0 | 82.3  (77.2-87.3)^22^ | 181/220 | 94.7  (93.6-95.7)^23^ | 1,699/1,795 | 65.3  (59.7-71.0) | 97.8  (97.1-98.5)^24^ |
| AFB/Xpert/T-SPOT^¶^ | TBAg/PHA≥0.5 | 83.6  (78.8-88.5)^25^ | 184/220 | 92.1  (90.9-93.4)^26^ | 1,654/1,795 | 56.6  (51.2-62) | 97.8  (97.1-98.6)^27^ |
|  | TBAg/PHA≥1.0 | 82.7  (77.7-87.7)^28^ | 182/220 | 93.8  (92.7-94.9)^29^ | 1,684/1,795 | 62.1  (56.6-67.7) | 97.8  (97.1-98.5)^30^ |
| A total of 2,044 patients had BALF and/or sputum AFB, culture, and Xpert assays, as well as peripheral blood mononuclear cell T-SPOT performed simultaneously. For strict comparison of the performance of AFB, Xpert, and T-SPOT, alone or in combination, only the first AFB, Xpert, and T-SPOT test results were used in the analysis. Pulmonary tuberculosis was defined as at least one of the BALF and/or sputum specimens having one positive culture result for *M. tuberculosis*. ^¶^Twenty-nine patients with invalid T-SPOT results (PHA spot forming cells <20) were excluded from the analysis, including 7 culture-confirmed MTB cases (1 AFB^+^/Xpert^-^, 4 AFB^-^/Xpert^+^, and 2 AFB^-^/Xpert^-^), 1 NTM cases with AFB^+^/Xpert^-^, and 21 culture-negative cases (1 AFB^-^/Xpert^+^ and 20 AFB^-^/Xpert^-^). PPV=positive predictive value; NPV=negative predictive value; CI=confidence interval; AFB=acid-fast bacilli smear; Xpert=Xpert MTB/RIF; T-SPOT=T-SPOT.*TB*; T-SPOT^MDC^=manufacturer-defined cutoff; TBAg=*Mycobacterium tuberculosis*-specific antigen; PHA=phytohaemagglutinin; BALF=bronchoalveolar lavage fluid. †Sensitivity comparison with Xpert: ^1^*p*<0.0001. ^4^*p*<0.0001. ^7^*p*<0.0001. ^10^*p*=0.907. ^19^*p*=0.349. ^22^*p*=0.494. Sensitivity comparison with AFB: ^13^*p*=0.001. ^16^*p*=0.063. Sensitivity comparison with AFB/Xpert: ^25^*p*=0.342. ^28^*p*=0.488. ^‡^Specificity comparison with Xpert: ^2^*p*<0.0001. ^5^*p*=0.005. ^8^*p*<0.0001 ^11^*p*=0.645. ^20^*p*=0.003. ^23^*p*=0.398. Specificity comparison with AFB: ^14^*p*<0.0001. ^17^*p*=0.024. Specificity comparison with AFB/Xpert: ^26^*p*=0.001. ^29^*p*=0.144. **^§^**NPV comparison with Xpert: ^3^*p*<0.0001. ^6^*p*<0.0001. ^9^*p*<0.0001. ^12^*p*=0.931. ^21^*p*=0.417. ^24^*p*=0.06. NPV comparison with AFB: ^15^*p*=0.108. ^18^*p*=0.338. NPV comparison with AFB/Xpert: ^27^*p*=0.423. ^30^*p*=0.515. | | | | | | | |
